# Combined Melatonin and Extracorporeal Shock Wave Therapy Enhances Podocyte Protection and Ameliorates Kidney Function in a Diabetic Nephropathy Rat Model

**DOI:** 10.3390/antiox10050733

**Published:** 2021-05-06

**Authors:** Chang-Chun Hsiao, You-Syuan Hou, Yu-Hsuan Liu, Jih-Yang Ko, Chien-Te Lee

**Affiliations:** 1 Graduate Institute of Clinical Medical Sciences, College of Medicine, Chang Gung University, Taoyuan 33302, Taiwan; cchsiao@mail.cgu.edu.tw (C.-C.H.); poo779779@gmail.com (Y.-S.H.); 498h007@stust.edu.tw (Y.-H.L.); 2 Center for Shockwave Medicine and Tissue Engineering, Kaohsiung Chang Gung Memorial Hospital and Chang Gung University College of Medicine, Kaohsiung 83301, Taiwan; kojy@cgmh.org.tw; 3 Division of Nephrology, Department of Internal Medicine, Kaohsiung Chang Gung Memorial Hospital and Chang-Gung University College of Medicine, Kaohsiung 83301, Taiwan; 4 Department of Orthopedic Surgery, Kaohsiung Chang Gung Memorial Hospital and Chang Gung University College of Medicine, Kaohsiung 83301, Taiwan

**Keywords:** melatonin, podocyte protection, extracorporeal shock wave, diabetic nephropathy

## Abstract

(1) Background: Diabetic nephropathy (DN) is common complication of diabetes. Current therapy for DN does not include promotion of podocyte protection. Therefore, we investigated the therapeutic effect of melatonin (Mel) combined extracorporeal shock wave (SW) therapy on a DN rat model. (2) Methods: The DN rats were treated with Mel (5 mg/kg) twice a week for 6 weeks and SW treatment once a week (0.13 mJ/mm^2^) for 6 weeks. We assessed urine microalbumin, albumin to creatinine ratio (ACR), glomerular hypertrophy, glomerular fibrosis, podocyte markers (Wilm’s tumor protein-1, synaptopodin and nephrin), cell proliferation, cell survival, cell apoptosis, renal inflammation and renal oxidative stress. (3) Results: The Mel combined SW therapy regimen significantly reduced urine microalbumin excretion (3.3 ± 0.5 mg/dL, *p* < 0.001), ACR (65.2 ± 8.3 mg/g, *p* < 0.001), glomerular hypertrophy (3.1 ± 0.1 × 10^6^ μm^3^, *p* < 0.01) and glomerular fibrosis (0.9 ± 0.4 relative mRNA fold, *p* < 0.05). Moreover, the Mel combined SW therapy regimen significantly increased podocyte number (44.1 ± 5.0% area of synaptopodin, *p* < 0.001) in the Mel combined SW group. This is likely primarily because Mel combined with SW therapy significantly reduced renal inflammation (753 ± 46 pg/mg, *p* < 0.01), renal oxidative stress (0.6 ± 0.04 relative density, *p* < 0.05), and apoptosis (0.3 ± 0.03 relative density, *p* < 0.001), and also significantly increased cell proliferation (2.0 ± 0.2% area proliferating cell nuclear antigen (PCNA), *p* < 0.01), cell survival, and nephrin level (4.2 ± 0.4 ng/mL, *p* < 0.001). (4) Conclusions: Mel combined SW therapy enhances podocyte protection and ameliorates kidney function in a DN rat model. Mel combined SW therapy may serve as a novel noninvasive and effective treatment of DN.

## 1. Introduction

Diabetic nephropathy (DN) is a common complication of diabetes mellitus [1,2]. The decline in renal function eventually progresses to end stage renal disease (ESRD) [3,4,5]. The pathogenesis of DN is inflammation [5,6], oxidative stress [6], and hyperglycemia [4]. Clinical manifestations of DN are glomerular hypertrophy, urine albumin excretion, glomerular fibrosis, and increased extracellular matrix (type I collagen and fibronectin) production [3,7]. Podocytes play a key role in preserving the glomerular filtration barrier integrity [8]. Podocyte foot processes prevent the urinary leakage of plasma proteins [9]. Thus, podocyte apoptosis or dysfunction not only leads to proteinuria but is also a key factor that drives glomerulosclerosis in the pathogenesis of DN [8,10]. Nephrin deficiency is considered a pathologic feature of glomerular injury [11]. Nephrin is required to maintain slit diaphragm integrity to preserve podocyte viability and glomerular structure and function in kidneys [11].

Melatonin (Mel) is mainly secreted by the pineal gland and has a renal protective effect [12]. Mel has been found to be a powerful antioxidant used to inhibit oxidative stress and the production of active oxidative substances [12,13,14,15]. Mel also has anti-inflammatory properties [12,13]. Oxidative stress and inflammation can cause podocyte damage or apoptosis [16]. Therefore, Mel might have a protective effect on podocytes. The main reason is that Mel has an anti-apoptotic effect on podocyte damage caused by oxidative stress and inflammation.

Extracorporeal shock wave (SW) acts through mechano-transduction at the cellular level in the body tissues [17,18,19]. The SW exerts its effects through other mechanisms, such as increased cell proliferation [17,18], anti-apoptotic [17,18], inhibition of oxidative stress [17,18,20], anti-inflammation [17,18,20], activating axonal regeneration [19], improved nerve regeneration [21,22], and promoted pancreatic beta cells regeneration [18]; and SW therapy polarizes M1 macrophages to anti-inflammatory M2 macrophages to inhibit inflammation [17,23].

Mel is anti-inflammatory and inhibits oxidative stress. SW inhibits oxidative stress, has anti-inflammation effects, has anti-apoptotic effects, increases cell proliferation, and promotes cell regeneration. Mel combined SW therapy may benefit the DN kidney. Our hypothesis is that Mel combined with SW therapy reduces oxidative stress, reduces inflammation and apoptotic, increases podocyte number, and reduces urine microalbumin excretion, glomerular hypertrophy and glomerular fibrosis in a DN rat model.

## 2. Materials and Methods

### 2.1. Animals

Wistar rats (250–300 g) were purchased from BioLASCO (Taipei, Taiwan). The animal center at Kaohsiung Chang Gung Memorial Hospital (CGMH) provided veterinary care to the Wistar rats for the care and use of experimental animals. All rats were housed at 22–24 °C under a 12-h light and dark cycle and were given food and tap water. Wistar rats were randomized to the normal group (*N* = 8), the DN group (*N* = 8), and the Mel combined SW group (*N* = 8). This study was approved by the Institutional Animal Care and Use Committee (IACUC: 2019092002) at CGMH.

### 2.2. Establishment of the DN Rat Model

The DN rat model was induced according to our previously published study [17]. DN was induced in the overnight fasted rats by a one-time intraperitoneal injection of streptozotocin (STZ) (50 mg/kg, Sigma-Aldrich, St. Louis, MO, USA) dissolved in citric acid buffer 40 mg/mL (pH 4.5, Sigma, St. Louis, USA) [17,24,25]. Rat blood glucose was maintained at 350 mg/dL by injection with insulin (0.4 unit/rat) and maintained for 12 weeks to establish the DN rat model successfully [17,26,27].

### 2.3. Mel Combined SW Treatment

Six weeks after injection of STZ, the DN rats received Mel (5 mg/kg) [28] injection via intraperitoneal twice a week for 6 weeks and the SW treatment once a week for 6 weeks. The SW treatment was performed according to our previously published protocols [17,18]. Briefly, ultrasound (Toshiba, Tokyo, Japan) was used to locate the kidneys. The source of SW was a EvoTron R05 (High Medical Technologies, Lengwil, Switzerland). EvoTron R05 (High Medical Technologies, Lengwil, Switzerland) was placed on the mark of kidney and a total of 200 impulses (0.13 mJ/mm^2^) was delivered.

### 2.4. Measurement of Urine Microalbumin and Creatinine

We collected 24-h urine samples from the normal group, the DN group, and the Mel combined SW group. Urine microalbumin levels were determined by microalbumin ELISA kit (Abcam, Cambridge, UK), and the urinary creatinine was measured by using a microplate assay kit (Abcam, Cambridge, UK) according to the manufacturer’s protocol.

### 2.5. Hematoxylin and Eosin (HE) Stain and Glomerular Volume

HE stain was performed according to our previously published protocols [17,18]. Kidney tissues were fixed with 4% paraformaldehyde and embedded in paraffin. Paraffin sections were stained with HE according to the manufacturer’s protocol. Mean glomerular volume was calculated according to the Weibel and Gomez formula [24,29].

### 2.6. Immunohistochemistry (IHC)

IHC was performed according to our previously published protocols [17,18]. Kidney tissue slides were heat sectioned in 10 mM citrate buffer with a pressure cooker. After that, the sections were incubated with primary antibodies for NOX4 (Abcam, Cambridge, UK), PCNA (Abcam, Cambridge, UK), fibronectin (Abcam, Cambridge, UK), CD68 (Abcam, Cambridge, UK), or collagen I (Abcam, Cambridge, UK) overnight at 4 °C. Slides were then probed with secondary antibody (Vector Laboratories, Burlingame, CA USA) for 1 h at room temperature. Slides were processed for color reaction with peroxidase treatment using 3,3′-diaminobenzidine substrate kit (SK-4100, Vector Laboratories, Burlingame, USA) and counterstained with hematoxylin. Ten glomeruli in each section were randomly selected for microscopy (Carl Zeiss, Gottingen, Germany). Six regions within glomeruli from three sections obtained from six rats were detected. Positive labeled and total cells in each section were counted, and percentage of positively labeled cells was calculated as percentage of the area (Image-Pro Plus software, Media Cybernetics, Silver Spring, MD, USA). 

### 2.7. Real-Time Quantitative Polymerase Chain Reaction (PCR) Analysis

Total RNA from the kidneys was isolated by using Trizol reagent (Invitrogen, Carlsbad, CA, USA) according to the manufacturer’s protocol. The TaqMan Reverse Transcription Kit (Applied Biosystems, Foster City, CA, USA) and a Gene Amp by Bio-Rad My Cycler (Bio-Rad, Hercules, CA, USA) were used to generate cDNA. Gene expression analysis was determined by quantitative real-time PCR using the SYBR Green Master Mix and a 7500 Real-time PCR System (Applied Biosystems, Foster City, CA, USA). The expressions of mRNA were normalized to the expression level of glyceraldehyde-3-phosphate dehydrogenase (GAPDH) mRNA and are relative to the average of all ΔCt (calculated by subtracting the Ct number of target sample from that of control sample) values in each sample using the cycle threshold Ct method.

### 2.8. Immunofluorescence (IF)

IF was performed according to our previously published protocols [17,18]. Kidney sections were blocked with 10% horse serum for 1 h. Kidney tissue slides were probed with primary antibodies CD206 (Abcam, Cambridge, UK), HO-1(Abcam, Cambridge, UK), synaptopodin (Abcam, Cambridge, UK), or F4/80 (Santa Cruz, Dallas, TX, USA) and incubated at 4 °C overnight. Kidney tissue slides were subsequently incubated with fluorescent secondary antibodies (Invitrogen, Carlsbad, CA, USA). Ten glomeruli in each section were randomly selected for the Olympus confocal microscope (Olympus, Tokyo, Japan). Six regions within renal glomeruli from three sections obtained from six rats were detected. Percentage of positive labeled cells was calculated as percentage of the area (Image-Pro Plus software, Media Cybernetics, Silver Spring, MD, USA).

### 2.9. Western Blot (WB)

WB was performed according to our previously published protocols [17,18]. Kidney tissue was dissociated with radioimmunoprecipitation assay (RIPA) lysis buffer and protein concentrations were determined. Fifty μg protein was separated by sodium dodecyl sulfate polyacrylamide gel electrophoresis (SDS-PAGE), transferred to polyvinylidene fluoride (PVDF) membrane (Millipore, Burlington, USA) and probed with primary antibodies NOX4 (Abcam, Cambridge, UK), WT-1 (Abcam, Cambridge, UK), Bax (Abcam, Cambridge, UK), pAKT (Abcam, Cambridge, UK), HO-1 (Abcam, Cambridge, UK) or CD68 (Abcam, Cambridge, UK) at 4 °C overnight. Horseradish peroxidase-conjugated IgG was the secondary antibody and visualized by chemiluminescence.

### 2.10. Enzyme-Linked Immunosorbent Assay (ELISA)

Kidney expression of nephrin, IL-6, IL-4 and IL-10 was determined using the Quantikine ELISA Kit in accordance with the protocol specified by the manufacture (R&D Systems, Minneapolis, MN, USA).

### 2.11. Terminal Deoxynucleotidyl Transferase dUTP Nick End Labeling (TUNEL) Staining

Apoptotic cell death was determined by using TUNEL staining (Roche Diagnostics, Basel, Switzerland) according to our previously published protocols [17,18].

### 2.12. Statistical Analysis

All experiments were repeated three times. All calculations were executed using SPSS statistical software (version 13.0, SPSS, Chicago, IL, USA). Results are expressed as mean ± standard error of the mean. Comparisons between groups were made by using one-way ANOVA (^‡^ *p* < 0.001, ^†^ *p* < 0.01, and * *p* < 0.05).

## 3. Results

### 3.1. Mel Combined SW Therapy Significantly Reduced Urine Microalbumin Excretion, Albumin to Creatinine Ratio (ACR) and Glomerular Hypertrophy in DN

**Figure 1:**
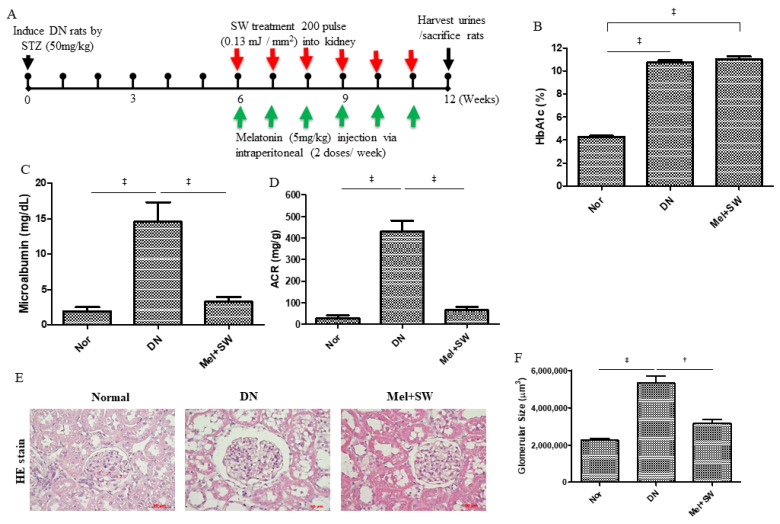
Melatonin (Mel) combined extracorporeal shock wave (SW) therapy improved renal function in diabetic nephropathy (DN). (**A**) Mel combined SW treatment protocol for DN rats. Red arrows indicate that DN rats received SW (200 pulse, energy density of 0.13 mJ/mm^2^) for 6 weeks. Green arrows indicate that DN rats had Mel (5 mg/kg) via an intraperitoneal injection (2 doses/week) for 6 weeks and then were killed at 12 weeks for study. (**B**) Blood hemoglobin A1c (HbA1c). Data are represented as mean ± SEM (*N* = 8); ‡, *p* < 0.001. (**C**) Urine microalbumin excretion. ‡, *p* < 0.001, (*N* = 8). (**D**) Albumin to creatinine ratio (ACR). ‡, *p* < 0.001, (*N* = 8). (**E**) Representative images of kidney tissue stained with hematoxylin and eosin (HE stain); bar = 50 μm. (**F**) Glomerular volume determined from hematoxylin and eosin (HE) stain sections. ‡, *p* < 0.001, †, *p* < 0.01 (*N* = 6).

Potential therapeutic effects of Mel combined with SW therapy on DN rats were evaluated using a treatment protocol (Figure 1A). The DN group had significantly increased blood hemoglobin A1c (HbA1c) compared with the normal (Nor) group; however, the Mel combined SW (Mel + SW) group had nearly the same blood HbA1c level as the DN group (Figure 1B). Urine albumin excretion reflects renal dysfunction of DN [3]. The DN group had significantly increased urine microalbumin excretion compared with the Nor group (Figure 1C). The Mel combined SW group had significantly reduced urine microalbumin excretion compared with the DN group (Figure 1C). The DN group had significantly increased albumin to creatinine ratio (ACR) compared with the normal group (Figure 1D). The Mel combined SW group had significantly reduced ACR compared with the DN group (Figure 1D). Glomerular hypertrophy is another clinical manifestation of DN [3,7]. Hematoxylin and eosin staining (HE stain) demonstrated that the DN group had significantly increased glomerular volume indicative of glomerular hypertrophy compared with the normal group (Figure 1E,F), whereas the Mel combined SW group prevented glomerular hypertrophy in the DN group (Figure 1E,F).

### 3.2. Mel Combined SW Therapy Significantly Reduced Glomerular Fibrosis in DN

**Figure 2:**
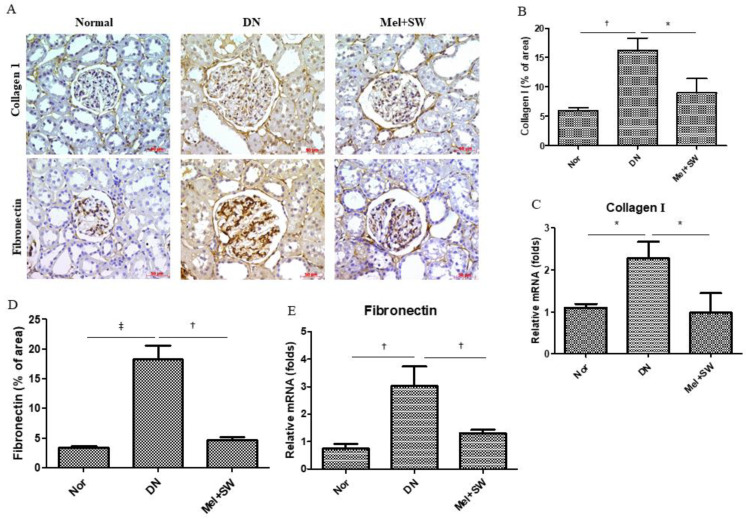
Mel combined SW therapy significantly reduced glomerular fibrosis in DN. (**A**,**B**,**D**) The glomerular fibrosis was determined by IHC staining detection for type I collagen (Collage 1) and fibronectin and quantification of IHC staining by image analysis; bar = 50 μm. (**C**,**E**) Real-time quantitative PCR analyses of collage 1 and fibronectin expression in renal tissue. †, *p* < 0.01, *, *p* < 0.05, ‡, *p* < 0.001. (*N* = 6–8).

Glomerular fibrosis is one of the clinical manifestations of DN [3,7]. Glomerular fibrosis significantly increased both type I collagen and fibronectin level compared with the normal group [17,30]. Immunohistochemistry (IHC) staining and real-time quantitative PCR analyses showed that the DN group had significantly elevated extracellular matrix production (type I collagen and fibronectin) in the kidneys compared with the normal group; however, the Mel combined SW group prevented renal fibrosis in the DN group (Figure 2A–E). The Mel combined SW therapy significantly reduced glomerular fibrosis in DN.

### 3.3. Mel Combined SW Therapy Significantly Enhanced Podocyte Number, Podocyte Viability and Glomerular Function

**Figure 3:**
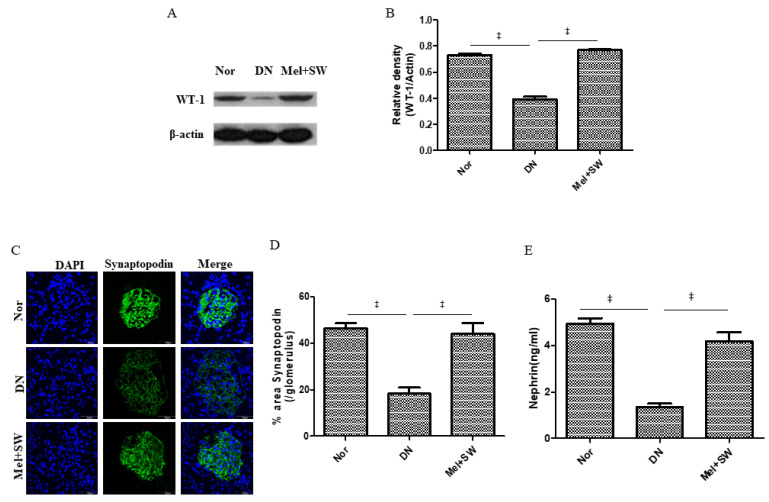
Mel combined SW therapy significantly increased podocyte number and enhanced podocyte viability and glomerular function. (**A**,**B**) Western blot (WB) analyzed WT-1 expression in renal tissue and quantification of WB by densitometric analysis. (**C**,**D**) Representative fluorescent images of glomeruli by IF stained with synaptopodin (green) indicating podocytes and quantification of IF stained by image analysis; bar = 50 μm. (**E**) ELISA analyzed nephrin level in renal tissue to represent the degree of preserved podocyte viability and glomerular function in kidneys. ‡, *p* < 0.001, (*N* = 6–8). Normal (Nor) group; 4’,6-diamidino-2-phenylindole (DAPI).

Progression of DN is related to podocyte injury and loss, whereas reversal of DN requires restoration of podocytes [8,10,31]. Wilm’s tumor protein-1 (WT-1) and synaptopodin are podocyte markers [31,32]. The DN group had a significantly decreased podocyte number compared with the normal group; however, the Mel combined SW group had a significantly enhanced podocyte number compared with the DN group (Figure 3A–D), suggesting that the Mel combined SW therapy significantly enhanced podocyte number in terms of DN. High nephrin levels are required to preserve glomerular function and podocyte viability in kidneys [11]. The DN group had significantly reduced nephrin level in the kidneys compared with the normal group; however, the Mel combined SW group was a significantly enhanced nephrin compared with the DN group (Figure 3E), suggesting that the Mel combined SW therapy significantly enhanced podocyte viability and glomerular function in kidneys.

### 3.4. Mel Combined SW Therapy Significantly Increased Cell Proliferation and Cell Survival and Significantly Reduced Cell Apoptosis

**Figure 4:**
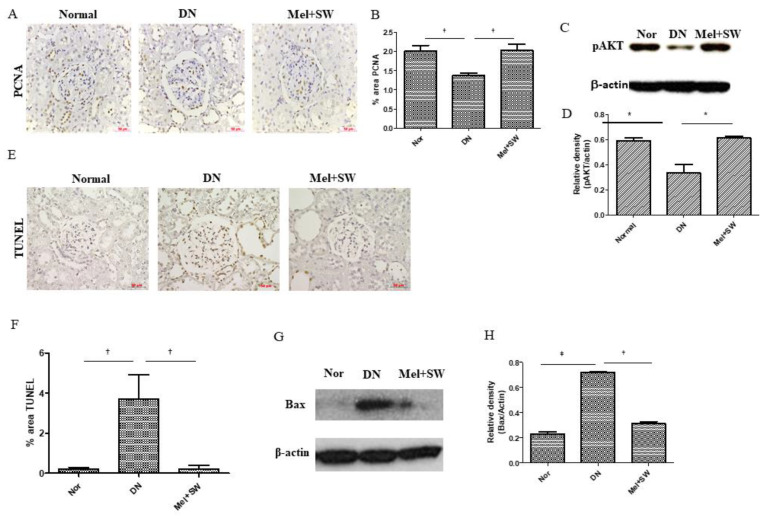
Mel combined SW therapy significantly increased cell proliferation and survival, and significantly reduced apoptosis. (**A**,**B**,**E**,**F**) Representative images of renal tissue IHC stained with PCNA for evaluation of cell proliferation, terminal deoxynucleotidyl transferase dUTP nick end labeling (TUNEL) indicating cell apoptosis and quantification of IHC staining by image analysis; bar = 50 μm. (**C**,**D**,**G**,**H**) Western blot (WB) analyzed _p_AKT and Bax expression in renal tissue and quantification of WB by densitometric analysis. _p_AKT evaluated cell survival, Bax evaluated cell apoptosis. †, *p* < 0.01, *, *p* < 0.05, ‡, *p* < 0.001, (*N* = 6–8).

The DN group showed significantly reduced cell proliferation in the kidneys compared with the normal group; however, the Mel combined SW group showed significantly increased cell proliferation in the kidneys compared with the DN group (Figure 4A,B). The DN group showed significantly reduced cell survival in the kidneys compared with the normal group, but the Mel combined SW group demonstrated significantly enhanced cell survival in the kidneys, more than the DN group (Figure 4C,D), therefore the Mel combined SW therapy significantly enhanced cell survival potency. The DN group showed significantly increased cell apoptosis compared with the normal group (Figure 4E–H). The Mel combined SW group showed significantly reduced cell apoptosis compared with the DN group (Figure 4E–H). The Mel combined SW therapy significantly increased cell proliferation and cell survival and significantly reduced cell apoptosis.

### 3.5. Mel Combined SW Therapy Significantly Reduced Renal Inflammation and Significantly Increased Renal Anti-Inflammation

**Figure 5:**
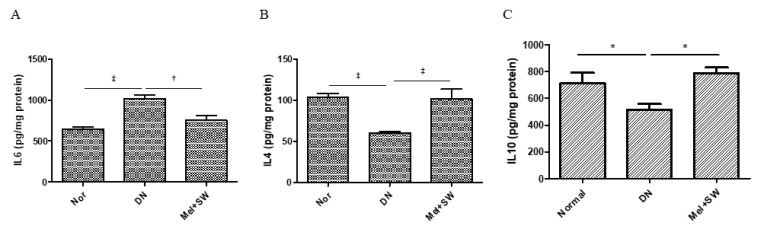
Mel combined SW therapy significantly reduced renal inflammation and significantly increased renal anti-inflammation. (**A**–**C**) ELISA analyzed IL-6, IL-4, and IL-10 expression in renal tissue. IL-6 indicates inflammation. IL-4 and IL-10 indicate anti-inflammation. ‡, *p* < 0.001, †, *p* < 0.01, *, *p* < 0.05 (*N* = 6–8).

Inflammation is a crucial pathogenetic mechanism in DN [5]. The DN group exhibited a high level of IL-6 expression in renal tissue, whereas Mel combined SW therapy significantly reduced the level of the inflammatory cytokine IL-6 (Figure 5A). Moreover, Mel combined SW therapy significantly increased the anti-inflammatory mediators IL-4 and IL-10 in the kidneys compared with DN group (Figure 5B,C). Mel combined SW therapy prevented diabetes-induced renal inflammation.

### 3.6. Mel Combined SW Therapy Significantly Reduced M1 Macrophages and Significantly Increased Anti-Inflammatory M2 Macrophages

**Figure 6:**
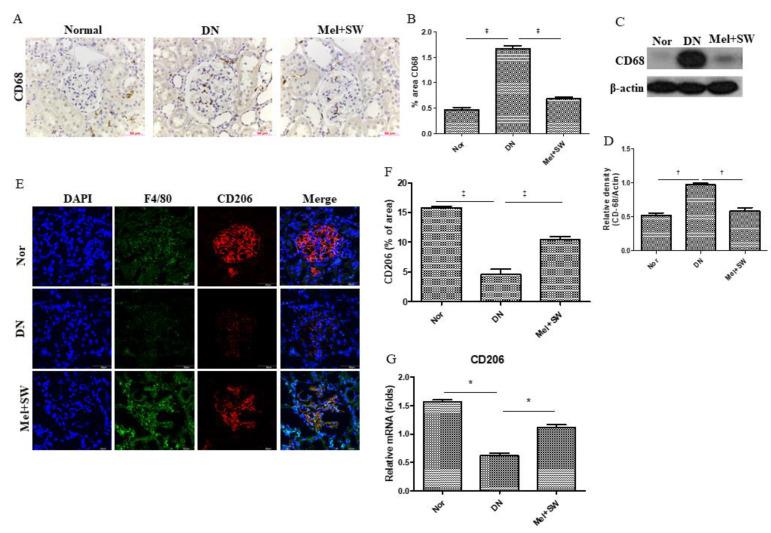
Mel combined SW therapy significantly reduced M1 macrophages and significantly increased anti-inflammatory M2 macrophages. (**A**,**B**) The number of M1 macrophages was determined by IHC staining detection for CD68 and quantification of IHC staining by image analysis; bar = 50 μm. (**C**,**D**) WB analyzed CD68 expression in renal tissue and quantification of WB by densitometric analysis. (**E**,**F**) Representative images of renal tissue IF stained with F4/80 (green); CD206 (red) indicating anti-inflammatory M2 macrophages and quantification of IF staining by image analysis; bar = 50 μm. (**G**) Real-time quantitative PCR analyses of CD206 expression in renal tissue. *, *p* < 0.05, †, *p* < 0.01, ‡, *p* < 0.001, (*N* = 6–8).

The DN group had significantly increased M1 macrophages in the kidneys compared with the normal group; however, the Mel combined SW group had significantly less M1 macrophages compared with the DN group (Figure 6A–D). CD206 is the marker of anti-inflammatory M2 macrophages [33]. Moreover, the Mel combined SW group had significantly increased anti-inflammatory M2 macrophages in the kidneys compared with the DN group (Figure 6E–G). These results suggested that Mel combined SW therapy might polarize M1 macrophages to anti-inflammatory M2 macrophages in order to inhibit inflammation.

### 3.7. Mel Combined SW Therapy Significantly Reduced Renal Oxidative Stress and Significantly Increased Renal Antioxidative Stress

**Figure 7:**
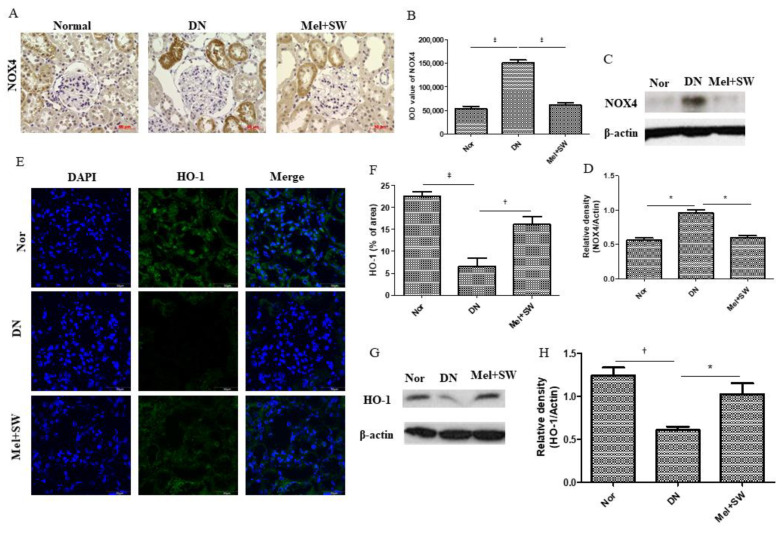
Mel combined SW therapy significantly reduced renal oxidative stress and significantly increased renal antioxidative stress. (**A**,**B**) Representative images of renal tissue immunohistochemistry (IHC) stained with NOX4 indicating oxidative stress and quantification of IHC staining by image analysis; bar = 50 μm. (**C**,**D**) WB analyzed NOX4 expression in kidney and used densitometric analysis to quantify WB data. (**E**,**F**) Representative IF stained with HO-1 (green) indicating antioxidative stress of glomeruli and used image analysis to quantify IF stained data; bar = 50 μm. (**G**,**H**) WB analyzed HO-1level in kidney and used densitometric analysis to quantify WB data. ‡, *p* < 0.001, *, *p* < 0.05, †, *p* < 0.01, (*N* = 6–8).

Oxidative stress is a crucial factor in the pathogenesis of DN [6]. The DN group had significantly increased oxidative stress level compared with the normal group (Figure 7A–D). The Mel combined SW group had significantly reduced oxidative stress level compared with the DN group (Figure 7A–D). The DN group had a significantly reduced level of antioxidative stress in the kidneys compared with normal group (Figure 7E–H). Moreover, The Mel combined SW group had a significantly increased level of antioxidative stress compared with the DN group (Figure 7E–H).

## 4. Discussion

Oxidative stress, inflammation and hyperglycemia are major factors in the pathogenesis of DN [4,5,6]. Inflammatory cytokines, mainly IL-6, are involved in the development and progression of DN [4]. Mel combined SW therapy polarized M1 macrophages to anti-inflammatory M2 macrophages in order to significantly increase the anti-inflammatory mediators IL-10 and IL-4 and significantly decrease the inflammatory cytokine IL-6. SW therapy significantly increases anti-inflammation in a DN rat model [17], in a rat model of acute myocardial infarction [23] as well as in a rat model of streptozotocin induced diabetes mellitus (DM) [18]. Fibroblast growth factor 1 (FGF1) therapy through an anti-inflammatory mechanism ameliorates kidney function in a DN model [34]. FGF1 was highly effective in preventing the activation of NF-kB in renal tissue of a DN mouse model [34,35]. Reactive oxygen species generated from NADPH oxidase (NOX) isoforms induce inflammation and apoptosis [36]. Our results show that activation of NOX4 occurred in kidney tissue of DN rats and, importantly, Mel combined SW therapy prevented NOX4 activation and significantly reduced apoptosis and oxidative stress. Low-energy SW therapy is also reported to alleviate oxidative stress and reduce apoptosis in a rat model of DN and DM [17,18]. Mel combined SW therapy did not change the hyperglycemia of DN rats. FGF1 significantly reduced blood glucose levels in db/db mice [34]. 

Clinical manifestations of DN are urine albumin excretion, glomerular hypertrophy, and glomerular fibrosis [3,7]. Podocyte apoptosis or dysfunction not only lead to proteinuria but are also key factors that drive glomerulosclerosis in the pathogenesis of DN [8,10]. Nephrin is required to maintain slit diaphragm integrity to preserve podocyte viability and glomerular structure and function in kidneys [11]. Glomerular parietal epithelial cells (PEC) contribute to adult podocyte regeneration [37,38,39]. Our results confirmed that there were significantly reduced podocyte numbers and nephrin level in the kidneys of DN rats and, importantly, Mel combined SW therapy significantly increased podocyte numbers and nephrin level in renal tissue of a DN rat model. Thus, Mel combined SW therapy significantly reduced urine microalbumin excretion, ACR and glomerular fibrosis in the DN rat model. CXCL12 blockade increased podocyte numbers and attenuated proteinuria in mice with adriamycin-induced nephropathy [40]. Low-energy SW therapy is also reported to significantly increase podocyte numbers and significantly reduce urinary albumin level in a rat model of DN [17]. Podocyte regeneration was further increased to 32.6% when the GSK3 inhibitor BIO was administered in a focal segmental glomerulosclerosis mice model [41]. SW therapy enhances beta cells number in a DM rat model [18]. 

The Mel combined SW group showed significantly decreased (near the normal group range) urine microalbumin compared with the DN group (Table 1). The SW therapy group showed significantly decreased (near the normal group range) urinary albumin level compared with the DN group [17] (Table 1). The Mel combined SW group showed significantly decreased ACR compared with the DN group, but there are no ACR data on the SW therapy [17]. The Mel combined SW group showed significantly decreased glomerular volume compared with the DN group. The SW therapy avoided glomerular hypertrophy in the DN group [17] (Table 1). The Mel combined SW group showed significantly decreased (near the normal group range) glomerular fibrosis compared with the DN group (Table 1). The SW therapy group had a significantly decreased glomerular fibrosis level compared with the DN group, but it was higher than the normal group level [17] (Table 1). The Mel combined SW group had significantly increased (near the normal group range) podocyte number compared with the DN group (Table 1). The SW therapy group had a significantly increased (near the normal group range) podocyte number compared with the DN group [17] (Table 1), but there are no synaptopodin data on the SW therapy [17]. The Mel combined SW group showed significantly increased podocyte viability compared with the DN group and lower viability compared to the normal group level. There are no podocyte viability data on the SW therapy [17]. Therefore, combined Mel and SW therapy shows synergetic effects compared with SW therapy only.

The Mel combined SW group showed significantly reduced (near the normal group range) oxidative stress levels compared with the DN group (Table 2). The SW group showed significantly reduced oxidative stress compared with the DN group, but the levels were higher than the normal group level [17] (Table 2). The Mel combined SW group had significantly increased (near the normal group range) antioxidative stress compared with the DN group (Table 2). The SW group had significantly increased antioxidative stress compared with the DN group [17] (Table 2). The Mel combined SW group had significantly reduced inflammation compared with the DN group, but it was higher than the normal group (Table 2). The SW group also had significantly reduced inflammation compared with the DN group, but it was higher than the normal group [17] (Table 2). The Mel combined SW group had significantly increased anti-inflammation compared with the DN group, and it was higher than the normal group (Table 2). The SW group had significantly increased anti-inflammation compared with the DN group, and it was higher than the normal group [17] (Table 2). The Mel combined SW group had significantly increased (near the normal group level) cell proliferation compared with the DN group (Table 2). The SW group had significantly increased cell proliferation compared with the DN group [17] (Table 2). The Mel combined SW group had significantly reduced cell apoptosis compared with the DN group (Table 2). The SW group had significantly reduced cell apoptosis compared with the DN group, but it was higher than the normal group level [17] (Table 2). Therefore, the Mel combined SW therapy showed synergistic effects compared with the SW therapy alone.

The Mel group showed significantly reduced urine microalbumin and inflammation compared with the DN group [42]. Mel therapy also decreased glomerular fibrosis and morphological changes in the kidney [42].

**Figure 8:**
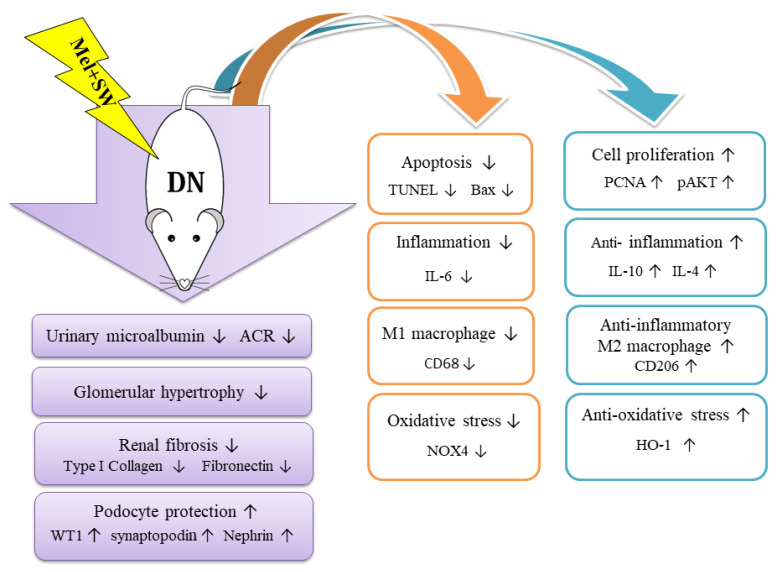
Proposed mechanisms of Mel combined SW therapy on the DN rat model.

SW therapy mechanisms of various diseases have been published, including increase of anti-inflammation [17,18,20,43,44] and anti-oxidative stress [17,18,20,45]. The mechanisms of Mel therapy in various diseases have also been published, including increase of anti-oxidative stress [15,46,47,48,49,50] and anti-inflammation [50,51,52,53]. Based on our findings, the proposed mechanisms of Mel combined SW therapy on DN are summarized in Figure 8.

## 5. Conclusions

The Mel combined SW therapy significantly reduced urinary microalbumin excretion and ACR, and significantly decreased glomerular hypertrophy and renal fibrosis in the DN rat model. Moreover, Mel combined SW therapy significantly enhanced podocyte regeneration, podocyte viability and glomerular function in DN. This was primarily attributed to the fact that Mel combined SW therapy significantly reduced renal oxidative stress and inflammation, significantly increased renal antioxidative stress and anti-inflammation, and significantly increased cell proliferation and cell survival, while significantly reducing cell apoptosis. Mel combined SW therapy is a novel noninvasive and effective treatment for DN.

## Figures and Tables

**Table 1:** Effect of Mel combined SW therapy and SW therapy on DN Rat Model.

	Mel Combined SW Therapy	SW Therapy ^§^
Urine Microalbumin	Mel + SW: 3.3 ± 0.5 ^‡^	SW: 2.3 ± 1.5 ^†^
(mg/dL)	Nor: 2.0 ± 1.4	Nor: 2.0 ± 1.4
	DN: 14.5 ± 2.7	DN: 6.2 ± 2.1
Glomerular volume	Mel + SW: 3.1 ± 0.1 ^†^	SW: 2.1 ± 0.4 ^‡^
(10^6^ μm^3^)	Nor: 2.3 ± 0.1	Nor: 2.4 ± 0.24
	DN: 5.3 ± 0.3	DN: 3.5 ± 0.4
Glomerular Fibrosis		
Type I collagen	Mel + SW: 90% *	SW: 111% ^‡^
(percent of Nor)	Nor: 100%	Nor: 100%
	DN: 230%	DN: 248%
Podocyte number		
WT-1	Mel + SW: 114% ^‡^	SW: 110% ^‡^
(percent of Nor)	Nor: 100%	Nor: 100%
	DN: 57%	DN: 48%

^§^ [17], ^‡^, *p* < 0.001VS DN, ^†^, *p* < 0.01 VS DN, *, *p* < 0.05 VS DN.

**Table 2:** Antioxidative stress and anti-inflammation effects of Mel combined SW therapy and SW therapy.

	Mel Combined SW Therapy	SW Therapy ^§^
Oxidative stress	Mel + SW: 107% *	SW: 130% ^‡^
(percent of Nor)	Nor: 100%	Nor: 100%
	DN: 171%	DN: 262%
Antioxidative stress	Mel + SW: 1.02 ± 0.14 *	SW: 3.0 ± 0.4 *
HO-1	Nor: 1.24 ± 0.1	Nor: 0.1 ± 0.04
Relative density	DN: 0.6 ± 0.06	DN: 0.5 ± 0.2
(HO-1/Actin)		
Inflammation	Mel + SW: 116% ^†^	SW: 130% *
IL6	Nor: 100%	Nor: 100%
(percent of Nor)	DN: 158%	DN: 172%
Anti-inflammation		
IL10	Mel + SW: 111% *	SW: 132% ^†^
(percent of Nor)	Nor: 100%	Nor: 100%
	DN: 72%	DN: 88%
Cell proliferation		
PCNA	Mel + SW: 100% ^†^	SW: 162% *
(percent of Nor)	Nor: 100%	Nor: 100%
	DN: 70%	DN: 96%
Cell apoptosis		
(percent of Nor)	Mel + SW: 130% ^‡^	SW: 114% *
	Nor: 100%	Nor: 100%
	DN: 304%	DN: 157%

^§^ [17], ^‡^, *p* < 0.001 vs. DN, ^†^, *p* < 0.01 vs. DN, *, *p* < 0.05 vs. DN.

## Data Availability

The data used to support the findings of this study are available from the corresponding author upon request.
